# Privacy-Preserving Vehicular Rogue Node Detection Scheme for Fog Computing

**DOI:** 10.3390/s19040965

**Published:** 2019-02-25

**Authors:** Basmah Al-Otaibi, Najla Al-Nabhan, Yuan Tian

**Affiliations:** 1 Department of Computer Science, King Saud University, Riyadh 12371, Saudi Arabia; 437202883@student.ksu.edu.sa; 2 School of Computer Engineering, Nanjing Institute of Technology, Nanjing 210044, China

**Keywords:** security, VANET, fog computing, rogue node detection, privacy preservation, authentication

## Abstract

In the last few decades, urban areas across the world have experienced rapid growth in transportation technology with a subsequent increase in transport-related challenges. These challenges have increased our need to employ technology for creating more intelligent solutions. One of the essential tools used to address challenges in traffic is providing vehicles with information about traffic conditions in nearby areas. Vehicle ad-hoc networks (VANETs) allow vehicle-to-vehicle (V2V) and vehicle-to-infrastructure (V2I) communication with the aim of providing safe and efficient transportation. Since drivers might make life-critical decisions based on information provided by other vehicles, dealing with rogue vehicles that send invalid data or breach users’ privacy is an essential security issue in VANETs. This paper proposes a novel privacy-preserving vehicular rogue node detection scheme using fog computing. The proposed scheme improves vehicle privacy, communication between vehicles, and computation efficiency by avoiding the exchange of traffic data between vehicles, allowing communication only through roadside units (RSUs). This scheme also proposes an RSU authentication mechanism, along with a mechanism that would allow RSUs to detect and eliminate vehicles providing false traffic data, which will improve the accuracy and efficiency of VANETs. The proposed scheme is analyzed and evaluated using simulation, which presents significant improvements for data processing, accurately detecting rogue vehicles, minimizing overhead, and immunizing the system against colluding vehicles.

## 1. Introduction

Vehicle ad-hoc networks (VANETs) represent the future of vehicle technology and intelligent transportation. VANETs provide vehicle-to-vehicle (V2V) and vehicle-to-infrastructure (V2I) communication that improves road safety, provides warning messages, increases comfort, and shares information (including media), among providing other services. Such features are available using vehicles’ ability to exchange safety messages between vehicles and the infrastructure, allowing drivers to avoid hazards and traffic congestion. Employing VANET to improve road safety is paramount today due to the increasing number of vehicles, number of traffic accidents, and death rate [1,2].

In VANETs, vehicles are able to communicate with each other to provide information about traffic and road conditions. This communication can be utilized to reduce road accidents and limit traffic congestion. However, it creates many security issues, since some vehicles can provide false information to other vehicles. Thus, false information should be detected and handled appropriately. The communication between vehicles may also violate vehicle privacy [3]. In addition to these security-related challenges, a vehicle needs to process the data it has collected and received from other vehicles. One of the possible alternatives is to send the collected traffic data to the cloud to perform the required computation and then communicate the results to vehicles, which can limit the computation and communication overhead between vehicles and improve their privacy. However, since road information is time-sensitive, this alternative solution might be inefficient.

**Figure 1:**
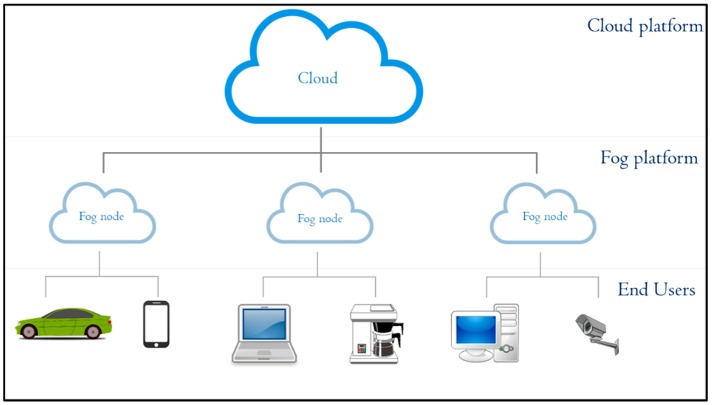
Overview of fog computing architecture.

Fog computing, which was introduced by Cisco Systems as a new computing paradigm, extends cloud computing by performing the most time-sensitive data computation and analysis at the edge of the network [4]. Fog computing is a virtualized platform providing storage, computing, and internet services to end users. In fog computing, fog nodes are located between end users and the cloud, as shown in Figure 1 [5,6]. Fog computing can be employed as an alternative to perform the calculation of road situation using road side units (RSUs) as fog nodes. In such scenarios, RSUs collect traffic data from vehicles within each RSU area. The collected data is analyzed by RSUs to extract the road situation. Communication between vehicles to detect the road situation can be done indirectly through the fog nodes. 

In this work, we propose a novel privacy-preserving vehicular rogue vehicle detection scheme using fog computing. The proposed scheme employs a model that uses fog nodes to perform computation needed by nearby vehicles and collaborate with other fog nodes. It also allows the detection of rogue vehicles that provide false traffic data in order to correct their data. Furthermore, this paper proposes a secure authentication scheme to authenticate the communication between RSUs.

The contribution of this paper is as follows:

- Providing an authentication scheme to secure communication between fog nodes.
- Employing fog computing to improve security in VANETs.
- Improving vehicle privacy by allowing the exchange of traffic data between vehicles through fog nodes.
- Detecting rogue vehicles and correcting their provided data.
- Protecting vehicles against collusion.
- Improving efficiency by reducing the computational overhead for a vehicle by executing the traffic calculation using fog nodes.

The rest of the paper is organized as follows: Section 2 discusses the existing related approaches in the literature. The proposed system is described in Section 3. Experimental results and analysis are presented in Section 4. In Section 5, we conclude our findings and highlight future work. 

## 2. Related Work

This section will review the most recent and relevant approaches to authentication in VANETs, rogue node detection techniques, and security issues in fog computing.

### 2.1. Authentication Scheme in VANET

Authentication is an important issue in VANETs to verify that the information comes from a legitimate source [7]. There are many proposed authentication schemes for VANETs, including symmetric key authentication and asymmetric key authentication [7,8]. Public key infrastructure (PKI) uses a public-private cryptographic key pair to secure data exchanged in the network. Authentication is achieved with a digital signature using PKI, where the message is signed with the sender’s private key and verified at the receiver side using sender’s public key. 

PKI has been used by many approaches in VANET [9,10] to authenticate and secure communication between vehicles. In [9] the authors propose a non-interactive ID-based scheme that uses member identity (ID) to establish a secure trust in V2V communication. They use a blind signature-based scheme to allow anonymous interaction between vehicles and RSUs. Using a symmetric key to secure the communication process between vehicles would reduce the high computational load caused by using the asymmetric key. Efficient conditional privacy preservation (ECPP) protocol has been proposed in [10]. ECPP uses a short time anonymous key between RSUs and on-board units (OBU). OBU will request a temporary public key certificate from an RSU in the area, but the OBU will first authenticate the RSU, then send a request with its real ID and its pseudo-ID to request short time anonymous keys. The RSU is responsible for issuing temporary public key certificates to vehicles. This approach reduces the storage of anonymous keys in each OBU.

Several schemes use symmetric keys for the authentication process [11,12]. In [11] the authors propose timed efficient stream loss-tolerant authentication (TESLA). TESLA is a broadcast authentication protocol used to authenticate the message with use of a symmetric key approach. The sender and receiver in TESLA need to be loosely time-synchronized. TESLA generates keys using a one-way chain by applying a one-way hash function; however, using a one-way chain is a primitive cryptography method. The sender sends a packet attached with a message authentication code (MAC) to the receiver. The receiver will store the packet for a period of time, after some time the sender will send the key needed to authenticate the previous message and accept the packet after verification. TESLA is used for authenticating multicasting and broadcasting messages in the wireless ad-hoc network. TESLA uses a symmetric key approach and does not provide non-repudiation. TESLA is vulnerable to attacks due to memory-based denial of service (DoS) [11,13], so a modified version called TESLA++ has been proposed in [12]. TESLA++ is a modified version from TESLA that provides fewer memory requirements on the receiver without sacrificing security, which prevents memory-based DoS. In TESLA++, the sender will first broadcast the MAC that has been computed with the current key along with the key index, then send the message with the key. The receiver will use the key index with time and the time associated with the start of the sender’s key chain. The receiver will check that the key is still known only by the sender. The sender will send the message with the key used to calculate the messages’ MACs. The receiver will trace the one-way key chain back to a trusted key to verify the key. The receiver will compare the computed MAC with the MAC stored in the memory to validate the message [12,13].

### 2.2. Rogue Nodes Detection

A malicious node acting like a legitimate node is known as a rogue node. The rogue node could be an access point (AP), Internet of Things (IoT) node, or fog node [14].

A vehicle that provides incorrect information is considered to be a rogue node, and the author in [15] proposes a threshold anonymous announcement (TAA) scheme to detect rogue vehicles. A vehicle in a TAA scheme will receive information from other vehicles, but the vehicle must check that this information was provided by different vehicles in order to accept the information. The TAA checks that the message was signed by different vehicles and that there is no two-message from the same vehicle. The vehicle needs to receive a number of messages that exceed a threshold value for the message to accept the message. TAA is based on direct anonymous attestation and one-time anonymous authentication. In [16], the authors propose a rule-enforced security technique for VANETs (REST-Net). REST-Net can detect false messages by monitoring the beacon message and process plausibility checks. REST-Net is a rule-based intrusion detection system that uses a rule to define incorrect behavior. REST-Net checks the validity of the message by checking the vehicle behavior before and after the message was sent. The message will be detected as false if the vehicle’s behavior does not match the message that was sent.

In [17], the authors detect a malicious packet injected into an in-vehicle controller area network (CAN) using a deep neural network structure. There are two phases: the training phase and the detection phase. In the training phase, each packet will be labeled as a normal packet or an attack packet based on the features that represent the statistical behavior that has been extracted from the CAN packet. In the detection phase, the system will extract the CAN packet features and classify the packet as a normal packet or not. The work in [18] detects the node that drops or duplicates the received packet. This approach depends on the vehicle being monitored by the verifier, where the verifier is the vehicle with the smallest value of distrust. The verifier monitors the number of packets that the vehicles receive and forward. The verifier will detect if the vehicle sent the same packet twice and if the vehicle forwarded a packet that must be forwarded. Each vehicle has a distrust value that is set initially when the vehicle joins the network, and once the distrust value exceeds a threshold value, the vehicle will be detected as a rogue vehicle. The distrust value will increase when the verifier observes abnormal behavior.

The work in [19] proposes a detection scheme that detects rogue vehicles based on the kind of message and subsequent behavior of the sending vehicle. Their work increases the computation cost since they need to keep track of the vehicle behavior. The work in [20] proposes an intrusion detection system (IDS) to detect rogue vehicles using statistical techniques. Vehicles collect data from other vehicles and calculate it to detect if there is a difference between the calculated value and received values to detect a rogue vehicle. The author in [21] proposes an efficient and light-weight intrusion detection mechanism for the vehicular network (ELIDV) to detect false alert generation attacks. A rogue vehicle is detected through the use of reputation scores managed by the RSUs. A vehicle’s reputation score is increased when it proposes a legitimate behavior and decreases when it misbehaves.

### 2.3. Security in Fog Computing

Fog computing has security and privacy issues that need to be taken into account when using fog node. According to [22], some of these issues are inherited from cloud computing, and the others are caused by the nature of fog computing. Security issues include authentication, privacy, detecting rogue nodes, and trust. This research focuses on authentication between fog nodes. Fog computing is a more interesting area to the researchers since it provides more advantages than cloud computing [23,24,25]. The work in [26,27] uses fog computing in an IoT environment, while [28,29] use fog computing in a VANET environment.

The author in [26] proposes mutual authentication between fog nodes and users at the edge of the network using blockchain technology and a secret sharing technique. A fog node would maintain a blockchain that allows a user to authenticate any fog node in the architecture (fog nodes would be able to establish mutual authentication with each other). The fog node authenticates the end user using a secret sharing mechanism. Users and fog nodes would mutually authenticate each other using the certification provided by a cloud broker and information in the blockchain without resorting to the cloud. Brokers provide the fog node with a verification parameter to verify the user. The broker verifies the certification of the fog node and provides it with the public key. The user must first register in the cloud, which will then provide new user credentials so the cloud can be used in the final authentication process at fog level. The user would authenticate the fog node by verifying the fog node public key with the validation public key provided by the broker. The fog node would authenticate the user by decrypting the user’s credentials, which were encrypted using the fog public key, and verifying them with polynomial interpolation using the value provided by the user and cloud broker. The authors in [27] propose a fog computing based security (FOCUS) system to protect IoT devices from cyber-attacks. The system contains four components: VPN, traffic analysis, challenge-response, and firewall. FOCUS systems authenticate communication between IoT devices by using a challenge-response technique. FOCUS will authenticate the sources that are marked at the traffic analysis phase as suspicious sources by sending a challenge (question) to the source, and if the source does not reply to the challenge, or replies with the wrong answer, the source will be blocked by the FOCUS firewall. Once the source replies with the correct answer, it will be marked as trusted and allowed to access the VPN server.

The work in [28] secures the communication between fog nodes (traffic lights) and vehicles using computational Diffie–Hellman (CDH) puzzles. The traffic light would generate a CDH puzzle and use location-based encryption (LBE) to encrypt it before broadcasting the puzzle to nearby vehicles. A vehicle needs to solve the puzzle in a negotiated amount of time, generate proof, and send the proof to the traffic light. The traffic light will verify the proof and run an efficient schedule algorithm. The authentication scheme in [29] occurs between RSUs (as fog node) and vehicles using a Schnorr signature and the challenge-response mechanism. The RSU authenticates vehicles using a Schnorr signature by sending a fresh challenge to a vehicle, the vehicle responds with an anonymized Schnorr signature, and the RSU will validate the response. In their work, vehicles can authenticate RSUs using Diffie–Hellman and the shared symmetric key between RSUs and vehicles, which will be updated by vehicles in each session to ensure security.

## 3. Privacy-Preserving Vehicular Rogue Node Detection Scheme for Fog Computing

### 3.1. System Overview

A vehicle network is a dynamic network that allows vehicles to communicate with each other and with RSUs located in their immediate area (better placed within their communication range) in order to exchange information about traffic, roads conditions, and services. Direct communication between vehicles may disclose vehicle privacy and increase overhead computation. The proposed system will provide information about road situation through the RSUs, which will preserve vehicle privacy and reduce the computation overhead for vehicles.

**Figure 2:**
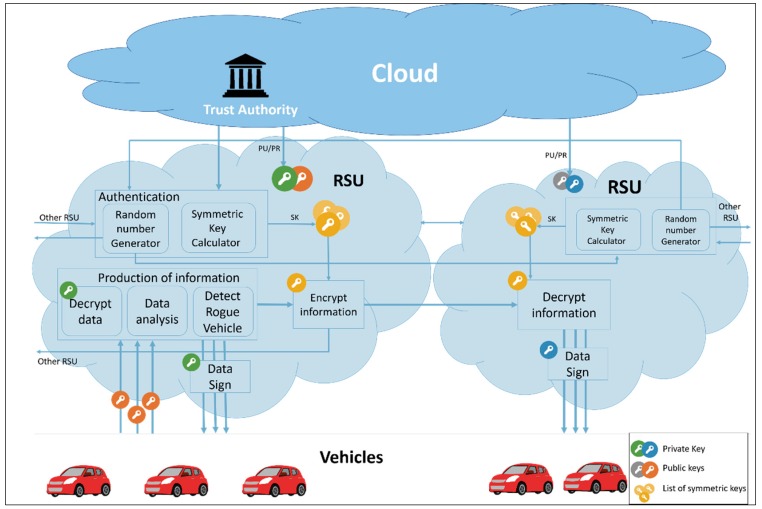
The system model of privacy-preserving vehicular rogue node detection scheme using fog computing.

In VANETs, communication must be secure, and the proposed scheme provides secure RSU-to-RSU communication. It uses the advantages of fog computing to reduce the communication overhead between end users (vehicles). In the proposed model, the RSUs represent fog nodes. Each vehicle is allowed to communicate with its nearest RSU(s). The vehicle provides the RSU with its traffic data, and the RSU will analyze the traffic data to get information about traffic and provide this information to other RSUs and vehicles. Furthermore, the RSU would detect a rogue vehicle providing false data about road conditions. Figure 2 shows the proposed system model.

Communication between RSUs will be encrypted using the symmetric key $K_{s}$. An RSU will inform other RSUs of their results about road situation, and this information will be encrypted using $K_{s}$, which will only be known by the RSUs authenticated by the trust authority. This data encryption process would guarantee that data comes from a legitimate entity.

### 3.2. Attacker Model

Adversary A’s goal is to intercept the communication between fog nodes F. A may acquire the encrypted message and a random seed. A will try to decipher the message to obtain the key used in the encryption process. However, if the message is deciphered and the key extracted, A will obtain the old key, and the fog nodes will be in different cycles with different keys. Furthermore, it is assumed that any fog node F will provide the other fog nodes with legitimate values. Finally, we assume no collusion occurs between the system parties that would result in disclosing the vehicle’s privacy, since vehicles are not part of the transmitted data.

### 3.3. Methodology Description

The system includes three main components: the trusted authority, RSUs, and vehicles. These components collaborate with each other to understand and realize the road condition in a secure manner. The trusted authority in the cloud will validate the system components and provide each RSU with the initial key $K_{m}\;$ used to create the symmetric key $\;K_{s}\;$, and provide public-private keys (pk,  sk) that will be used at the communication process with vehicles. Vehicles will know the RSU public key (pk) once they enter the area of the RSU and will use the pk in encrypting the vehicle message that will be sent to the fog node, while the private key will be used to sign traffic information before providing it to the vehicles, assuming that a vehicle is capable of encrypting and verifying the messages using fog node public keys. After a particular time, the trusted authority will check the validation information of the fog nodes and vehicles and provide them with new keys to use in their communication. The initial key $K_{m}$ will be the same for all RSUs, and each RSU have a symmetric key that is different from all other RSU symmetric keys but will be known for all other RSUs. 

Vehicles will communicate with RSUs by sending message P  containing the vehicle’s speed and location to the RSU in their area. This message will be encrypted using the RSU public key ${\left[P\right]}_{pk}$ to ensure that only authenticated vehicles will provide information to the RSU. The RSU will receive the message and decrypt it using its private key ${\left[C\right]}_{sk}$ and store the message to process it.

Each RSU will generate a random seed r that will be used with the authentication key $K_{m}$ to create their symmetric key $\;K_{s}$, and any invertible function can be used to combine the two numbers $K_{s}^{i}=\left[K_{auth}^{j},r_{i}\right]$. Each RSU will send their random seed r to other RSUs. Once the RSU receives r from other RSUs, it will calculate the $K_{s}\;$ locally for each RSU and store it with their random seed and ID. Each RSU will have a list of ($id,\;r,K_{s}$) for other RSUs. After calculating the symmetric key for all RSUs, the authentication key will be hashed to use the hashed authentication key as an authentication key for the next cycle $K_{auth}^{j}=\left[K_{auth}^{j-1}\right]$. After a designated period of time, each RSU will update its key table by calculating the new symmetric keys using the stored random seeds with the new authentication key $\;K_{s}^{i}=\left[K_{auth}^{j},r_{i}\right]$. A new RSU joining the network will be authenticated using the challenge-response technique. One of the RSUs in the network will send a challenge to the new RSU to reach the same cycle that the other RSUs are in, where the new RSU can gain the shared key if it responds correctly. The key generation algorithm is shown in Algorithm 1, the notation that has been used is listed in Table 1. The key-creating steps are as follows:

1. Initial key provided by the trust authority $\;K_{auth}^{0}=K_{m}$.
2. RSU generates a random seed $r_{i}$.
3. Symmetric key calculated using the authentication key and the random seed generated $K_{s}^{i}=\left[K_{auth}^{0},r_{i}\right]$.
4. Each RSU sends its random seed to other RSUs in the network $r_{i}$.
5. Each RSU will locally calculate $K_{s}\;$ for other RSUs.
6. Each RSU will store $r_{i}\;$ with the calculated $K_{s}^{i}$.
7. The initial key will be hashed and will used in the next cycle $K_{auth}^{1}=\left[K_{auth}^{0}\right]$.
8. After a designated period of time, each RSU will update its symmetric key and the stored list of keys by recalculating the symmetric key using the random seed and the hashed key $K_{s}^{i}=\left[K_{auth}^{j},r_{i}\right]$.
9. The hashed key will be hashed again to use it in the next cycle $K_{auth}^{j}=\left[K_{auth}^{j-1}\right]$.

**Algorithm 1.** Symmetric key generation at RSUx.$\mathrm{Input}:\;\mathrm{Initial}\;\mathrm{Key}\;K_{m}$, random seed for other RSU $1.\;K_{auth}^{j}$ = $K_{m}$ $2.\;r_{x}$ = GenerateRandomSeed() 3. **for** all RSU in the network  4.  $r_{i}$ = RecievingRandomSeed from RSU i 5.  StoreRandomSeed($r_{i}$, i) 6. **end** $7.\;K_{s}^{j}=\left[K_{auth}^{j},r_{x}\right]$ 8. **for** all RSU in the network  9.   $K_{s}^{i}=\left[K_{auth}^{j},r_{i}\right];$ 10.   StoreSymmetricKey($K_{s}^{i}$, i); 11. **end**
 12.$\;K_{auth}^{j+1}=\mathrm{Hash}\left[K_{auth}^{j}\right]$; 13. **if** (timeout) 14.   Go to step 7

Vehicles will provide data to the nearest RSU so it can process the data, detect rogue vehicles, extract the expected driving speed, and provide the vehicle with the expected driving speed and road situation. 

Each road has its predefined values such that the road speed is S. If the vehicle speed comes between these values [S−s, S+s] such that the s is a predefined small number, the speed can be considered to be a normal speed if it’s balanced with the number of vehicles. The rogue vehicle tends to provide a very high or very low speed when compared with legitimate vehicles, such that comparing vehicles’ speeds will help detect rogue vehicles.

The RSU will count the number of vehicles providing the data to estimate the expected speed on the road (if the number of vehicles providing the data is close to the number of vehicles that the road can absorb then the vehicles are expected to provide a speed close to the lower bound). The RSU will sort the received vehicles’ speeds in increasing order to help in the detection of rogue vehicles and correct their values. Each value will be compared with the two values that follow it, and RSUs will discard any values where the difference between them is greater than the threshold value. If the value is from the first half, the small values and all the previous value are detected as false information, since they represent a low speed when the road has no traffic jam. If the value is from the second half, the high value and all the following values are detected as false information, since this value represents an increase in speed during a traffic situation, or they are exceeding the speed limit. The system will calculate the mean speed value by summation all the speeds provided (the rogue vehicle speed is zero) and dividing them by the number of cars that provided the information (the rogue vehicle will be counted since it takes space in the road), then substitute the rogue vehicle’s speed with the mean speed to calculate the real mean speed (if the rogue vehicle has provided correct data).

When the RSUs analyze the information provided by the vehicles in their area and detect the road situation, the RSUs will use their symmetric key $K_{s}\;$ to encrypt the message and send it to other RSUs. When an RSU receives the encrypted message from another RSU, the RSU will look in its list of keys to find the key for this RSU, decrypt the message, and announce the received information to vehicles in their range after signing it.

If a new RSU wants to join the network, the trusted authority will verify the new RSU and provide pk, sk, and $K_{m}$. The new RSU will send their random seed $r_{x}\;$ to the other RSUs and then one of the RSUs (as verifier) in the network sends a challenge for the new RSU; the challenge is to encrypt the message using random seed $r_{x}\;$ and inform the new RSU that we are in cycle j. If the new RSU has the correct initial key, then they can easily encrypt the message correctly. The response to the challenge will contain the encrypted message; the verifier will decrypt the message using the new RSU’s key that was calculated locally using the new RSU’s random seed. If it is correctly decrypted and matches the message in the challenge, then the new RSU will join the network. The verifier will start a timer when sending a challenge, and if a timeout occurs, the new RSU needs to ask again to join the network.

### 3.4. System Implementation

There are three RSUs with respective IDs i (1,2,3), and the trusted authority has verified each RSU and provided each RSU the pk, sk, and $K_{m}$. The keys for each RSU are shown in Appendix A (see Table A1). The public key will be known to the vehicles in the RSU’s area. 

Each RSU i  will generate a $r_{i}\;$ to use to create the symmetric key $K_{s}^{i}$; in this case, the following Equation (1) will be used to generate the symmetric key.

(1) $$K_{s}^{i}=r_{i}mod\;K_{m}$$

Table 2 shows the random seed values in the second column. After choosing a random seed, each RSU will calculate its symmetric key locally $K_{s}^{i}$ using $r_{i}\;$ and $K_{m}$. RSU1 will send its random seed to RSU2 and RSU3, which equals 783,346. RSU2 will send its random seed, 368,263, to RSU1 and RSU3, and RSU3 will send its random seed, 294,958, to RSU1 and RSU2. RSU1 will store the random seeds from each RSU with the corresponding ID; each RSU will have a record of each RSU containing (i, $r_{i}$). In this case, we will have two records at RSU1 (2, 368,263) and (3, 294,958). The records in RSU2 will be (1, 783,346) and (3, 294,958). RSU3 will have the following records (1, 783,346) and (2, 368,263).

Each RSU will calculate $K_{s}^{i}$ locally for other RSUs using Equation (1), for example, in RSU1 the calculating for the RSU2 key will be as follows:

(2) $$K_{s}^{i}=r_{i}mod\;K_{m}\;K_{s}^{i}=783,346\;mod\;33\;\;K_{s}^{i}=25.$$

The resulting symmetric key for each RSU will be stored in the same record as its ID and random seed; each record will have (i, r, $K_{s}^{i}$).

The authentication key will be hashed $K_{auth}^{2}=\left[K_{auth}^{1}\right]$, and the new authentication key will be $K_{auth}^{2}=$ 00340034. In the next round, after 1 min, each RSU will update its key table by recalculating the symmetric key using the new authentication key $K_{auth}^{2}$. After updating the table, the authentication key will be hashed to use it in round three $K_{auth}^{3}=\left[K_{auth}^{2}\right]$. Table 3 shows the key table for RSU1, RSU2, and RSU3, respectively, after being updated. 

The vehicles in RSU1’s area will send their speeds to RSU1. At time $t_{0}$, there are 16 vehicles, and each vehicle will send its speed, after encrypting it, using RSU1 pk. The messages before and after encryption using RSA algorithm are shown in Appendix A (see Table A2).

RSU1 will receive the messages and decrypt them using its sk in order to extract the original message. It will order the vehicles’ speed in increasing order and count how many vehicles send a message to use this information later; the counter will equal 16 in this case. Each value will be compared with the following two values, as shown in (Table A3) in Appendix A, by taking the difference between them. If the difference is more than or equal to a threshold value (where the value is 20 in this case), the value and all the values less than it if the value is in the first half, will be detected as false information. If a value exceeds the threshold value and is in the last half, as in this case, it will be detected as false information, as well as all the values that are higher than it. The value that exceeds the threshold in this case is provided from vehicle 14. 

The remaining value will be used to calculate the road situation by taking their average value, which is equal to 37.38462. The vehicles’ speed table will be updated, as shown in Table 4. The detected value will be compensated for by substituting the average value, since these vehicles still take up space on the road. A new average value will be calculated (here, average value is 37.38462). Based on predefined road information and the calculated average value, the road situation will be detected.

After receiving the data and extracting the road situation, which, in this case, indicates that there is low traffic, the information will be encrypted using the RSU1 symmetric key and RC4 encryption algorithm as shown:

(3) C=E(Low Traffic,25)C=U2FsdGVkX18cUb5M4NTaIxpzZ1k/3ny2iuMG

and sent to RSU2 and RSU3. RSU1 will send this information to the vehicles in its area after signing it using its private key.

RSU2 and RSU3 will look in their key table to extract RSU1’s symmetric key to be able to decrypt the message as shown:

(4) P=D(U2FsdGVkX18cUb5M4NTaIxpzZ1k/3ny2iuMG,25)P=Low Traffic.

RSU2 and RSU3 will sign the message with their private keys and propagate it to vehicles in their areas, in this case, that RSU1’s area has low traffic.

## 4. Experiment Results and Analysis

In the simulation, we used a 5 km highway road with three lanes. The road’s speed was between 30 and 70 km/h. The data was collected from different vehicles; there were 3000 vehicles in the cognition situation, and the expected speed of the vehicles was between 20 and 40 km/h. The RSU was in the center and covered the entire road. The experiment was conducted using an HP laptop with operating system Windows 10 Pro Process Intel^®^ core™ i7-6500U CPU @2.50GHz 2.95GHz, installed memory (RAM): 16 GB, system type: 64-bit operating system, and ×64-based processor. 

The evaluation metrics used to evaluate the proposed work are the processing time, scalability, overhead, and detection rate, and there is a difference observed between the mean speed provided from the vehicles and the mean speed that the RSU reached after processing the data.

The metrics used are described in the following:

- Processing time: This is the time needed to process all the data provided.
- Scalability: This is the capability of the system to process data as the number of vehicles increases.
- Overhead: This indicates the additional messages exchanged on the network in order to detect the rogue vehicle.
- Detection rate: This is the percentage of rogue vehicles detected and classified as rogue vehicles.

**Figure 3:**
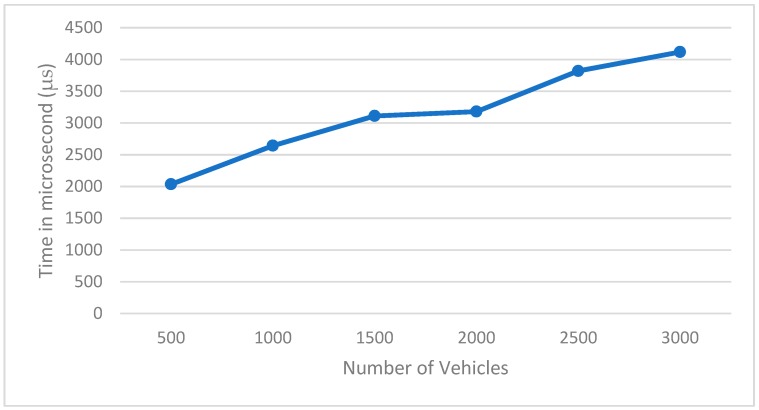
Data processing time.

Figure 3 shows the processing time (in microseconds) needed with the increasing number of vehicles. The maximum number of vehicles is 3000, since this is the maximum number of vehicles that the road can achieve. This evaluation shows that the system is scalable and it can handle a high number of vehicles in a brief time.

**Figure 4:**
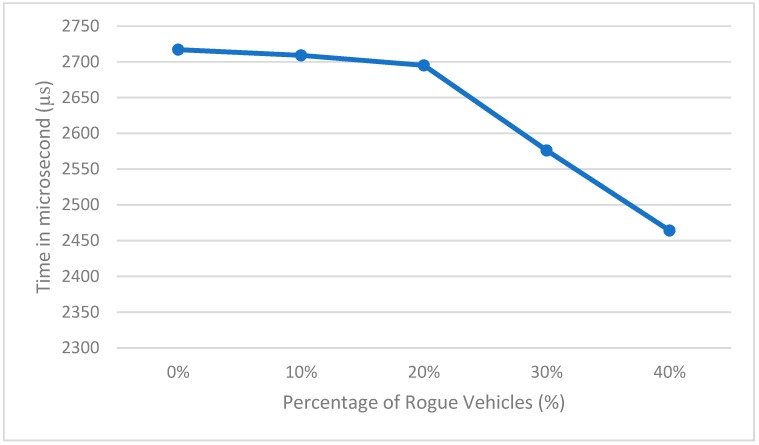
Data processing time with different percentage of rogue vehicles.

Figure 4 shows the processing time needed to process the data with different percentages of rogue vehicles when considering 1000 vehicles and the rogue vehicles providing high speeds. The results show that the processing time is decreased with an increasing number of rogue vehicles, since the system will not analyze the remaining data once a rogue vehicle has been detected (since the data is sorted in increasing order). The system time complexity for detecting rogue vehicles is *O*(*n*), where *n* is the number of vehicles that provide data to the RSU. The system will sort the provided data using any fast sorting algorithm with time complexity less than *O*(*n*), and will compare each value with the following two values to take the difference between them to detect rogue vehicles. 

**Figure 5:**
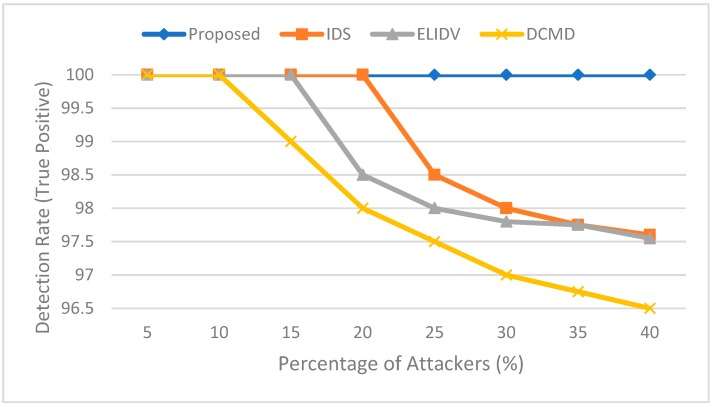
Detection rate comparison in case of false information attack.

The system is compared with the work in [19,20,21] in terms of detection rate. As shown in Figure 5, the proposed system detects all rogue vehicles and classifies them correctly since the system sorts the data in increasing order and compares the provided data with each other to detect the false data. The rogue vehicles provide high or low values that result in a difference between the values provided from legitimate vehicles exceeding the threshold values, and since our system sorts the provided data in increasing order, the data provided from rogue vehicles will be at the start or the end of the array, making it easy to detect and correct. Data centric misbehavior detection scheme (DCMD) in [19] is used to detects the rogue vehicle based on the subsequent behavior of the vehicle, IDS in [20] makes the decision based on the data collected from other vehicles, and ELIDV in [21] bases a decision on vehicle reputation and the collected score. The compared methods could result in decreasing the detection rate, since they are based on monitoring the behavior after providing the data or reputation scores, while the proposed method provides a high detection rate, since it compares the data provided from rogue vehicles with the data provided from legitimate vehicles. 

**Figure 6:**
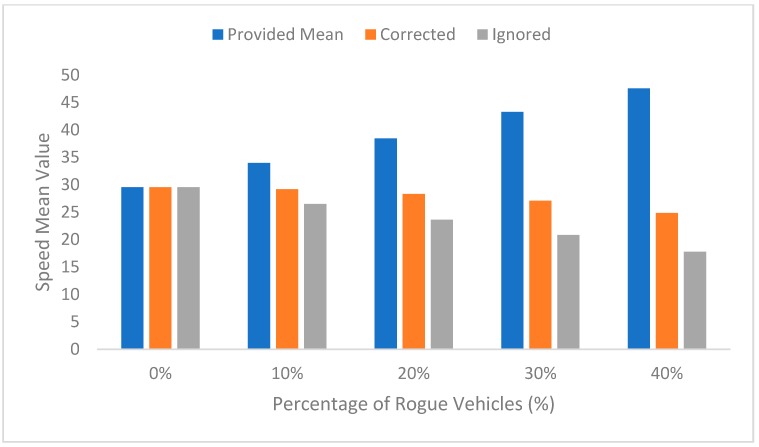
Varying of the speed mean value.

The difference between the mean speed provided from the vehicles (including rogue vehicles) and the mean speed that the RSU reached after processing the data after the false data had been corrected or ignored is shown in Figure 6. The increasing difference can be seen between the actual mean, the corrected mean, and the mean when ignoring the data provided from rogue vehicles when the percentage of rogue vehicles is increased. This difference could result in a critical situation if the system is not able to detect and correct the false data. It can be observed that there is no difference in the mean when there is no rogue vehicle, since all provided data is used. Ignoring data provided by rogue vehicles will decrease the final speed and will generate false information by increasing the rogue node percentage. This system provides the information after correcting the data, since the provided information will be based on the corrected speed.

Table 5 shows a comparison of different rogue vehicles’ detection schemes. In real scenarios, vehicles are not trusted and may collude to gather information about another vehicle by continuing to communicate with it. In this approach, vehicles communicate with RSUs to understand road conditions. Furthermore, this approach does not trace the movement of vehicles, which makes it immune to collusion, while other work does not protect against colluding vehicles. There is no extra overhead in this system, as it can detect rogue vehicles using the provided data with no need to exchange more messages. This system uses RSUs as infrastructure to detect rogue vehicles, while the work presented in [19,21] uses RSUs and vehicles as infrastructure for detection, and the work in uses the OBU in the vehicles. This system employs a fog computing paradigm to enhance security in VANETs by representing RSUs as fog nodes. The proposed system corrects the false data, since it affects the overall decision; ignoring it may cause a result that does not reflect the real information.

## 5. Conclusions

VANETs provide communication between V2V and V2I to improve road safety and efficiency. This work proposes a privacy-preserving vehicular rogue node detection scheme for fog computing. The proposed scheme allows information on road conditions to be shared between RSUs and nearby vehicles in a secure manner. It also preserves vehicle privacy and immunity from colluding vehicles. In the proposed work, a fog computing based model was designed to perform the required calculation of road situation at fog nodes represented by RSUs. Traffic data is collected by vehicles and provided to fog nodes in a secure manner. This scheme eliminates communication between vehicles to improve security and communication efficiency. It also detects rogue vehicles and eliminates incorrect data from the road situation calculation. The resulting calculation of traffic conditions is encrypted and shared with other fog nodes using symmetric key encryption. It is also signed and sent to nearby vehicles.

Experimental results and analysis show that the proposed scheme is scalable, efficient in terms of communication and computation overhead, and capable of detecting all rogue vehicles. In future, we plan to study different attack models, extend our model, and explore its practicality to improve the efficiency of the provided road services.

## Figures and Tables

**Table 1:** Notation table.

Symbol	Description
$K_{s}^{i}$	Encryption key for RSU *i*
M	Signed message
$\left[K_{auth}\right]$	Hash of authentication key
$K_{auth}^{j}$	Authentication key used in round *j*
$K_{m}$	Initial key provided by the trust authority
$r_{i}$	Random seed for RSU *i*
pk	Public key

**Table 2:** RSU1(a), 2(b) and 3(c) key table.

(a)			(b)			(c)		
i	$r_{i}$	$K_{s}^{i}$	i	$r_{i}$	$K_{s}^{i}$	i	$r_{i}$	$K_{s}^{i}$
1	783,346	25	2	368,263	16	3	294,958	4
2	368,263	16	1	783,346	25	1	783,346	25
3	294,958	4	3	294,958	4	2	368,263	16

**Table 3:** RSU1(a), 2(b) and 3(c) key table in round 2.

(a)			(b)			(c)		
*i*	$r_{i}$	$K_{s}^{i}$	i	$r_{i}$	$K_{s}^{i}$	i	$r_{i}$	$K_{s}^{i}$
1	783,346	10,3278	2	368,263	28,229	3	294,958	294,958
2	368,263	28,229	1	783,346	103,278	1	783,346	103,278
3	294,958	294,958	3	294,958	294,958	2	368,263	28,229

**Table 4:** Vehicles Speed after Modification.

i	Speed	i	Speed
1	25	9	40
2	28	10	43
3	29	11	44
4	32	12	45
5	35	13	47
6	39	14	37.38462
7	39	15	37.38462
8	40	16	37.38462

**Table 5:** Comparison with other misbehavior detection schemes.

Scheme	Immune from Collude	Overhead	Infrastructure	Response Mechanism	Fog
DCMD ^1^ [19]	No	Yes	Hybrid	Fine Imposition	No
IDS ^2^ [20]	No	Yes	OBU-based (cooperative)	Isolation	No
ELIDV ^3^ [21]	No	Yes	Hybrid	Ignore	No
Our Work	Yes	No	RSU-based	Correction	yes

^1^ Data centric misbehavior detection scheme, ^2^ Intrusion detection system, ^3^ Efficient and light-weight intrusion detection mechanism for the vehicular network.

## Abbreviations

The following abbreviations are used in this manuscript:

AP	Access Point
CAN	Controller Area Network
CDH	Computational Diffie–Hellman
DCMD	Data centric misbehavior detection
DoS	Denial of Service
ELIDV	Efficient and Light-weight Intrusion Detection mechanism for the Vehicular network
FOCUS	Fog computing based security
ID	Identity
IDS	Intrusion Detection System
IoT	Internet of Thing
LBE	Location-based encryption
MAC	Message Authentication Code
OBU	On-Board Unit
PK	Public Key
PKI	Public Key Infrastructure
REST-Net	Rule-Enforced Security Technique for VANETs
RSU	Road Side Unit
SK	Private key
TESLA	Timed Efficient Stream Loss-tolerant Authentication
V2I	Vehicle to Infrastructure
V2V	Vehicle to Vehicle
VANET	Vehicle Ad hoc Network

## Appendix A. System Implementation Data Details

**Table A1:** RSU keys provided from Trust Authority.

i	pk	sk	$K_{m}$
1	MFswDQYJKoZIhvcNAQEBBQADSgAwRwJAVxrbiSEL5Sf1EcUHQkhU34LNCl4zqXZq j4MQ2E8sJuizmJLnAOdyEq/yIlDOOVYPMgbt5s4f95/oWCnzZyUQFQIDAQAB	MIIBOQIBAAJAVxrbiSEL5Sf1EcUHQkhU34LNCl4zqXZqj4MQ2E8sJuizmJLnAOdy Eq/yIlDOOVYPMgbt5s4f95/oWCnzZyUQFQIDAQABAkAiqgSOCQGz23fy72cZILHu FR7GLoD+wqpbnHw6qR9YCDJw6PA08zu+02Kmsy85+eGFeNz2D+sM3LmsGXdT4DfN AiEAmcoWls0nbt0eypnuRPWKHEZzy0X4vLJjSVhKByNQmpcCIQCQ/v2Nxr5n4JAW 9BK8faEFQJLnvs++Iqii4N2uRrhcMwIhAIa+js4wEAXNzaW7+w0Giay+ecQ3mXlT XzSrG6lnYr8fAiAdt6VQAYPU1nmxuqR8bWMrKGjzhnAdkAzwFRZaObRfcQIgM6h5 EUkax05llb8IGNhMFWokdDIenpdm1F/B0Sknl8I=	33
2	MFswDQYJKoZIhvcNAQEBBQADSgAwRwJAWWxbte1ThfwfcuQwf6lG7B504fPfSwCp wjnA7iAi7a+MSyIglooy/v8SZZpz7hmPP9Fw2FAWJaLSwXbeNBbyEwIDAQAB	MIIBOQIBAAJAWWxbte1ThfwfcuQwf6lG7B504fPfSwCpwjnA7iAi7a+MSyIglooy /v8SZZpz7hmPP9Fw2FAWJaLSwXbeNBbyEwIDAQABAkBGa5jFagHub5/sgFrZDdt2 Mn3lOoHLtNf6xjRy0gfvmPK1nJilOC+ZYL1FC+QDYYY9zQIg1H8FFXs5+0rRAhrR AiEAnK/LzUO0cxMSmA5kSMZ9YF2Sq4bBxc9oph+kI/MK/J0CIQCSGk3P92SfG0Xb 4XLloAm83DebxacyAHixMXrtOXPybwIgGUmh+bnQmLXeTV4dP0WRnIjdkANKqLMl r5Hxur+R6V0CID9Zun2/ltjKmZsDAbABmddTYaVgqeOrgqnKe7PbIqRvAiEAgvBi zVjHaH9MZKR/RCXsJlAzeKPR//uEeJfQnat+Ja4=	33
3	MFwwDQYJKoZIhvcNAQEBBQADSwAwSAJBAO4zKT34b1MRSoFeyaOmPo6yOFS8n88K Fehb6Ac27P+Ff7Ru3KXV5Scr4yDEO+tsP36Oj/poIPJQFA4gExe0GzUCAwEAAQ==	MIIBPQIBAAJBAO4zKT34b1MRSoFeyaOmPo6yOFS8n88KFehb6Ac27P+Ff7Ru3KXV 5Scr4yDEO+tsP36Oj/poIPJQFA4gExe0GzUCAwEAAQJBAKStAgoxwuTuw0+FNGnK +Ny2IXOTo/gCxPqK73JtapOLZizEbDbGgNZmNkivYU2yKS4OrdawBdAU6/62lYsO 41ECIQD7LxO22yyyQpyR8ciOrZgyFAwKC+/L0yQ9f/Ju6Ir8mwIhAPLEWkK5rB3S 3mTI4rbRddAqnjW2nOASQC2vDEfw2PxvAiEAjWtJ7C+mEI8UW88HHd16zOcgiB+E WPt9ceqxceQXLHUCIQDrBmr3xDc8HESPv+e04+2x3UCTcbpIN4MIdzplf2ciYwIh AKk9Vn9MnEmeAnJXxSI1hN2Xs20L9Wt3pM4eFE2v/cWa	33

**Table A2:** Vehicles’ message.

Vehicle	*M* = Speed	Encrypt(M,sk)
1	40	S7r99OjaxGi9G78qyGSRnhzVnEubv4FCBvAmg0lZ07ljRlVj6aD3wxLI79FgV0yoeguMktQeo+QUnPacF3Evqw==
2	44	J3ZUSkJ0hoj+tZTMDHuVWWcoNTPRUszBTwufydCM7J/3mLGp1otbT8tgV0yUiV40Ps+uUTxplAm6AmT/5/tKWA==
3	80	Kv9Xs3Me7RvhFWqeqd4W9I+AVJ4syLo4FYq9n/59Csfv5GTSiFnOe24fM/yvM9mGz3VSBI0k73bsljzQCYz5Bg==
4	39	M7eEHp4RO9TQ/xA+mjfEFmRD3edzmRBvxP2I2ShBCiXkDI4w+CFcvNoXdGzB6OsQ9ss0eAtazUTY4vJ0+dg3xw==
5	25	Tkn+G+Wb1O1Y5HdgECh8XvRP2ocLFaCaH9igGWZgixGUM6vF5TFcODnwVWvuJW70hfNCNmOGXwsNmKi11w66dw==
6	35	Rb/ifHWfAdvkAtU+bpgmfcuyIDhYBSkpmORhUCwBfMgGAwW5z7FYEfw+TPb6TQGKUGJNXOJ5hw5B3wKkdK7fLQ==
7	120	HlgqAh2fYHW7++j6MHfAbeccjQxXsSUvg9KwWmmdWVlmE6I6X1XhC1QoIYv0X85fyuH860hVUe4bvrbptK9tFA==
8	40	KCAvJCosWzl/0OsgWczV0aWby1PjjL8jhAbROX6XNXv+clFZOPEcqqRJW6ohdyV/9BQu4kJz9b7kdlGGb8v+hw==
9	50	PKbN2c6V6LgCi2dCXz//ZWgJ+Hw2A7SrtcMSvQ8HH7l3mbX8FWhXsgqdybXdtodKGjP8l4wx2ltY7jm74fbUrQ==
10	45	FpmWe9LQFp9Pas5teIi+YAVdLIPA8WintYtAR7WUOAgnKb8ZeKRB6NtIzKeFe4g+KqL29kFw+PTiV8z8V66JHQ==
11	47	OfLLW6HEGClz2I/D9ZoIOmSeBu2VI4y0sf5YbhMjEzPpOjD0wyf6HdfcT7jpV4qlWUhx7uKiim53os5YMsOiDA==
12	39	J/GXfMcg+rAZff5lgSJhl3St+KxtSVjOFBwDIqI+sbKr0FPNAlV7vrlWPKSAKeTxJQnvho7bOwFhHHJZMZfMMg==
13	43	RFDsPBRdAF+oaa7QIvsaZmrLpXfFhRzL8T7nDF47ha5TNf7voK51CnZIivwJsoNv1K7kBHcfG8jRXFUWVD65Mg==
14	29	LP++KX+ZBi3yZjGhIaUTARHQUi6cg3ZnA1PVFAsynXC3Nc1uKG/Q8y02YEuMEv68/7MrFOZ9HOnCkpb8Cufrxg==
15	32	IMcpySaMUm+/QLFa907slM5uzeFARhRoBPr8awosLGzX/WWzHmBTPDHGrRuMpd3GKbkBNTFziA8Sf7zltm5jcw==
16	28	Pnw4wBBXliV+WKVOEnt3IsG0ivvO0sfgLlp0H51UTgbA31YYxVheGJW9cHuLt60ZHb8mjx36WB7T5ixu7Y9svQ==

**Table A3:** Vehicles’ speed.

i	Speed	$v_{i+1}-v_{i}$	$v_{i+2}-v_{i}$
1	25	3	4
2	28	1	4
3	29	3	6
4	32	3	7
5	35	4	4
6	39	0	1
7	39	1	1
8	40	0	3
9	40	3	4
10	43	1	2
11	44	1	3
12	45	2	34
13	47	32	33
14	79	1	41
15	80	40	-
16	120	-	-

## References

[1] Zaidi K., Milojevic M., Rakocevic V., Rajarajan M. Data Centric Rogue Node Detection in VANETs. Proceedings of the 2014 IEEE 13th International Conference on Trust Security and Privacy in Computing and Communications.

[2] Engoulou R.G., Bellaïche M., Pierre S., Quintero A. (2014). VANET security surveys. Comput. Commun..

[3] Pathan A.S.K. (2011). Security in Vehicular Ad Hoc Networks. Security of Self-Organizing Networks: MANET, WSN, WMN, VANET.

[4] Khan S., Parkinson S., Qin Y. (2017). Fog computing security: A review of current applications and security solutions. J. Cloud Comput..

[5] Law Y.W., Palaniswami M., Kounga G., Lo A. (2013). WAKE: Key management scheme for wide-area measurement systems in smart grid. IEEE Commun. Mag..

[6] Flavio B., Rodolfo M., Zhu J., Addepalli S. Fog computing and its role in the internet of things. Proceedings of the first edition of the MCC workshop on Mobile cloud computing.

[7] Riley M., Akkaya K., Fong K. (2011). A survey of authentication schemes for vehicular ad hoc networks. Secur. Commun. Netw..

[8] Zeadally S., Hunt R., Chen Y.-S., Irwin A., Hassan A. (2012). Vehicular ad hoc networks (VANETS): Status, results, and challenges. Telecommun. Syst..

[9] Li C.-T., Hwang M.-S., Chu Y.-P. (2008). A secure and efficient communication scheme with authenticated key establishment and privacy preserving for vehicular ad hoc networks. Comput. Commun..

[10] Lu R., Lin X., Zhu H., Ho P.-H., Shen X. ECPP: Efficient conditional privacy preservation protocol for secure vehicular communications. Proceedings of the INFOCOM 2008. The 27th Conference on Computer Communications.

[11] Perrig A., Canetti R., Tygar J.D., Song D. (2002). The TESLA broadcast authentication protocol. Rsa Cryptobytes.

[12] Studer A., Bai F., Bellur B., Perrig A. (2009). Flexible, Extensible, and Efficient VANET Authentication. J. Communic. Netw..

[13] Vaibhav A., Shukla D., Das S., Sahana S., Johri P. (2017). Security Challenges, Authentication, Application and Trust Models for Vehicular Ad Hoc Network—A Survey. IJ Wirel. Microw. Technol..

[14] Alrawais A., Alhothaily A., Hu C., Cheng X. (2017). Fog Computing for the Internet of Things: Security and Privacy Issues. IEEE Internet Comput..

[15] Chen L., Ng S.-L., Wang G. (2011). Threshold anonymous announcement in VANETs. IEEE J. Sel. Areas Commun..

[16] Tomandl A., Fuchs K.-P., Federrath H. REST-Net: A dynamic rule-based IDS for VANETs. Proceedings of the 2014 7th IFIP Wireless and Mobile Networking Conference (WMNC).

[17] Kang M.J., Kang J.W. (2016). Intrusion detection system using deep neural network for in-vehicle network security. PloS ONE.

[18] Daeinabi A., Rahbar A.G. (2013). Detection of malicious vehicles (DMV) through monitoring in Vehicular Ad-Hoc Networks. Multimed. Tools Appl..

[19] Sushmita R., Cavenaghi M.A., Huang Z., Nayak A., Stojmenovic I. On data-centric misbehavior detection in VANETs. Proceedings of the 2011 IEEE Vehicular technology conference (VTC Fall).

[20] Zaidi K., Milojevic M.B., Rakocevic V., Nallanathan A., Rajarajan M. (2016). Host-Based Intrusion Detection for VANETs: A Statistical Approach to Rogue Node Detection. IEEE Trans. Vehicular Technol..

[21] Sedjelmaci H., Senouci S.M., Abu-Rgheff M.A. (2014). An efficient and lightweight intrusion detection mechanism for service-oriented vehicular networks. IEEE Internet Things J..

[22] Yi S., Qin Z., Li Q. Security and Privacy Issues of Fog Computing: A Survey. Proceedings of the 10th International Conference on Wireless Algorithms, Systems, and Applications.

[23] Alrawais A., Alhothaily A., Hu C., Xing X., Cheng X. (2017). An attribute-based encryption scheme to secure fog communications. IEEE Access.

[24] Huang Q., Yang Y., Wang L. (2017). Secure Data Access Control with Ciphertext Update and Computation Outsourcing in Fog Computing for Internet of Things. IEEE Access.

[25] Wang Q., Chen D., Zhang N., Qin Z., Qin Z. (2017). LACS: A Lightweight Label-Based Access Control Scheme in IoT-Based 5G Caching Context. IEEE Access.

[26] Imine Y., Kouicem D.E., Lounis A., Bouabdallah A. MASFOG: An Efficient Mutual Authentication Scheme for Fog Computing Architecture. Proceedings of the 2018 17th IEEE International Conference on Trust, Security and Privacy in Computing and Communications/12th IEEE International Conference on Big Data Science and Engineering (TrustCom/BigDataSE).

[27] Alharbi S., Rodriguez P., Maharaja R., Iyer P., Bose N., Ye Z. FOCUS: A fog computing-based security system for the Internet of Things. Proceedings of the 2018 15th IEEE Annual Consumer Communications & Networking Conference (CCNC).

[28] Liu J., Li J., Zhang L., Dai F., Zhang Y., Meng X., Shen J. (2018). Secure intelligent traffic light control using fog computing. Future Gener. Comput. Syst..

[29] Wei J., Wang X., Li N., Yang G., Mu L. (2018). A Privacy-Preserving Fog Computing Framework for Vehicular Crowdsensing Networks. IEEE Access.

